# Clinically amyopathic dermatomyositis associated with anti-nuclear matrix protein 2 antibody

**DOI:** 10.1093/rap/rkab104

**Published:** 2021-12-20

**Authors:** Saori Abe, Hiroto Tsuboi, Hirofumi Toko, Fumika Honda, Mizuki Yagishita, Shinya Hagiwara, Yuya Kondo, Risa Konishi, Mari Okune, Yuki Ichimura, Naoko Okiyama, Isao Matsumoto

**Affiliations:** 1 Department of Internal Medicine; 2 Department of Dermatology, Faculty of Medicine, University of Tsukuba, Ibaraki, Japan

Key message

- Testing for myositis-specific antibodies could help in the diagnosis of atypical idiopathic inflammatory myopathies, such as clinically amyopathic DM associated with anti-nuclear matrix protein 2 antibodies.

Dear Editor, In recent years, a number of novel myositis-specific antibodies (MSAs) have been identified to classify clinical subsets of idiopathic inflammatory myopathies (IIMs) [1], although for diagnosis, anti-Jo-1 antibody is the only stated antibody in the EULAR/ACR classification criteria [2]. The anti-nuclear matrix protein 2 (NXP2) antibody, positive in 1.6–3.0% of adults with IIMs [3], is known to be associated with s.c. oedema and a severe muscle phenotype [1, 4–6]. Here, we report a rare case of clinically amyopathic DM (CADM) presenting with the anti-NXP2 antibody.

**Fig. 1:**
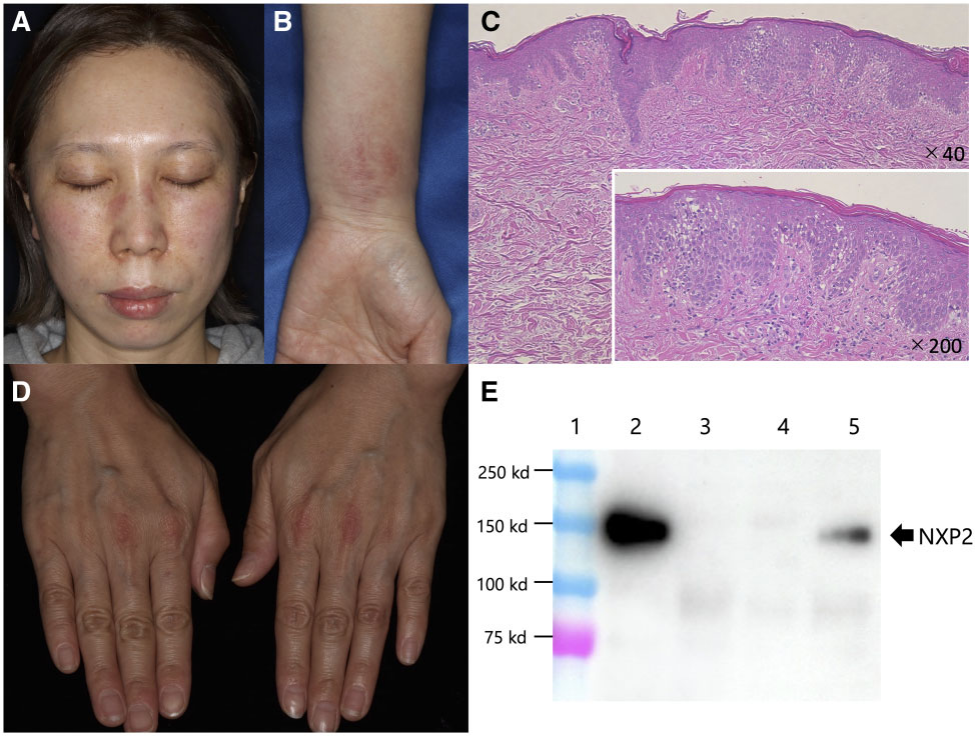
Skin involvements and detection of anti-nuclear matrix protein 2 antibody in the present case (**A**) Facial rash. The patient’s left cheek was more swollen than the right one. (**B**) Subcutaneous oedema with rash on the left forearm. (**C**) The skin biopsy findings showed basal cell vacuolization, a feature of interface dermatitis. (**D**) The Gottron sign, which appeared several months later than her forearm s.c. oedema. (**E**) Band image of immunoprecipitation followed by western blot analysis (IP-WB) for detection of anti-NXP2 antibody. Lane 1, molecular weight marker (Precision Plus Protein Standards Dual Color, Bio-Rad); lane 2, anti-NXP2 antibody-positive serum detected by use of radioisotope-immunoprecipitation and IP-WB; lane 3, anti TIF1-γ antibody-positive serum; lane 4, anti-MDA5 antibody-positive serum; lane 5, sera from this patient. The arrow indicates the molecular weight of NXP2. MDA5: melanoma differentiation-associated protein 5; NXP2: nuclear matrix protein 2; TIF1-γ: transcriptional intermediary factor 1-γ.

A 38-year-old woman, with a history of photosensitivity, presented with s.c. oedema and a rash that had been worsening over several months. The physical examination revealed lesions on her cheeks and left forearm, but not on the proximal limbs or the trunk (Fig. 1A and B). At the initial presentation, rashes were not found on her hands, nor was nailfold erythema. The laboratory test findings, such as CRP, creatinine kinase and eosinophil levels, were within normal limits. The IIF findings for ANAa and anti-cytoplasmic antibodies were negative, as were the disease-specific antibodies, including: the EIA findings for anti-aminoacyl transfer RNA synthetase (ARS) antibody; the ELISA findings for the anti-melanoma differentiation-associated gene 5 (MDA5), anti-transcriptional intermediary factor 1-γ (TIF1-γ) and anti-Mi-2 antibody; the chemiluminescent enzyme immunoassay findings for anti-DNA antibody; and the double immunodiffusion method findings for anti-RNP and anti-SSA antibody; all the laboratory tests used were commercially available ones. The chest X-ray did not show any abnormalities. MRI of the most significant oedematous lesion showed fasciitis in the forearm but no sign of inflammation in any muscle. Examination by a dermatologist concluded that the rashes were not disease specific, such as heliotrope rash or the Gottron sign/papule, but biopsy of the skin lesion showed interface dermatitis with increased dermal mucin, a finding corresponding to DM (Fig. 1C). Thus, we also performed electromyography and MRI of the proximal limbs; the results of both were normal. At that time, we could not make a definite diagnosis. During the 3 months after her first visit, the s.c. oedema continued to worsen, findings suggestive of oedematous myositis [7]. New rashes had also appeared, which were eventually determined to be the Gottron sign (Fig. 1D). Again, suspecting CADM, we additionally conducted tests for anti-small ubiquitin-like modifier-1 activating enzyme (SAE) and anti-NXP2 antibodies in the laboratory by immunoprecipitation followed by western blot analysis, which revealed positivity for the anti-NXP2 antibody (Fig. 1E). Finally, because of the delayed onset of the Gottron sign, we were able to diagnose CADM according to the EULAR/ACR IIMs criteria. Soon after the diagnosis was confirmed, the patient was given systemic glucocorticoids and the s.c. oedema improved rapidly. The disease was well controlled throughout the course, and no evidence of internal malignancy has occurred since the start of treatment.

The diagnosis of IIMs, especially CADM, can sometimes be challenging. This case highlights two important points related to the diagnosis of IIMs. First, it focuses on the utility of testing for MSAs. Knowing that anti-NXP2 antibody is significantly associated with severe muscular manifestation [4–6] and given the rarity of the clinically amyopathic phenotype [3, 6], we initially could not predict an anti-NXP2 antibody association in our patient, which led to our delayed testing for the antibody. Second, this case highlights a weakness of the present classification criteria. Even if we had known the patient’s positivity for the anti-NXP2 antibody soon after her arrival, we could not have diagnosed CADM at that point because her typical DM rash appeared much later. Such cases are not rare according to a previously reported external performance validation of the classification criteria for IIMs, showing that several DM patients could not be diagnosed correctly on the basis of the criteria, although they were positive for MSAs [8]. Another study also showed that 25% of patients with skin-predominant DM fail to meet the present classification criteria, thus calling for a revision [9]. These studies point out the weakness of the present criteria, which might have to be reconsidered in terms of placing more emphasis on MSAs. Furthermore, the frequency of MSAs and the associated clinical phenotype among diverse ethnic backgrounds must also be discussed. The frequency of MSAs is known to vary according to ethnic background, especially for anti-MDA5 and anti-TIF1γ antibodies [10]. The frequency of anti-NXP2 antibody also differs, having been reported in 1.6% in a Japanese cohort and in 11% in a Stanford University cohort [3, 6]. It remains unknown whether the associated clinical phenotype, such as the controversially reported risk of malignancy, differs among diverse ethnicities [1, 3, 6]. For a better understanding of the heterogeneity of MSAs, investigation in a highly diverse population is required.

In conclusion, we have reported a case of CADM associated with anti-NXP2 antibody, which has shed light on the value of testing for MSAs.
